# Targeting aurora kinases as a potential prognostic and therapeutical biomarkers in pediatric acute lymphoblastic leukaemia

**DOI:** 10.1038/s41598-020-78024-8

**Published:** 2020-12-04

**Authors:** Caroline Aquino Moreira-Nunes, Felipe Pantoja Mesquita, Adrhyann Jullyanne de Sousa Portilho, Fernando Augusto Rodrigues Mello Júnior, Jersey Heitor da Silva Maués, Laudreísa da Costa Pantoja, Alayde Vieira Wanderley, André Salim Khayat, William J. Zuercher, Raquel Carvalho Montenegro, Manoel Odorico de Moraes-Filho, Maria Elisabete Amaral de Moraes

**Affiliations:** 1 Pharmacogenetics Laboratory, Drug Research and Development Center (NPDM), Federal University of Ceará, Coronel Nunes de Melo st, n 1000, Rodolfo Teófilo, Fortaleza, CE CEP: 60416-000 Brazil; 2 Molecular Biology Laboratory, Ophir Loyola Hospital, Belém, PA Brazil; 3 Laboratory of Human Cytogenetics, Institute of Biological Sciences, Federal University of Pará, Belém, PA Brazil; 4 Department of Pediatrics, Octávio Lobo Children’s Hospital, Belém, PA Brazil; 5 Oncology Research Center, João de Barros Barreto University Hospital, Federal University of Pará, Belém, PA Brazil; 6 Division of Chemical Biology and Medicinal Chemistry, University of North Carolina at Chapel Hill, Eshelman School of Pharmacy, Chapel Hill, NC USA

**Keywords:** Haematological cancer, Cancer genetics, Gene expression, Cancer, Cell biology, Genetics, Molecular biology

## Abstract

Aurora kinases (AURKA and AURKB) are mitotic kinases with an important role in the regulation of several mitotic events, and in hematological malignancies, *AURKA* and *AURKB* hyperexpression are found in patients with cytogenetic abnormalities presenting a unfavorable prognosis. The aim of this study was evaluated the mRNA expression profile of pediatric Acute Lymphoblastic Leukaemia (ALL) patients and the efficacy of two *AURKA* and *AURKB* designed inhibitors (GW809897X and GW806742X) in a leukemia cell line as a potential novel therapy for ALL patients. Cellular experiments demonstrated that both inhibitors induced cell death with caspase activation and cell cycle arrest, however only the GW806742X inhibitor decreased with more efficacy *AURKA* and *AURKB* expression in K-562 leukemia cells. In ALL patients both *AURKA and AURKB* showed a significant overexpression, when compared to health controls. Moreover, *AURKB* expression level was significant higher than *AURKA* in patients, and predicted a poorer prognosis with significantly lower survival rates. No differences were found in *AURKA* and *AURKB* expression between gene fusions, immunophenotypic groups, white blood cells count, gender or age. In summary, the results in this study indicates that the *AURKA* and *AURKB* overexpression are important findings in pediatric ALL, and designed inhibitor, GW806742X tested in vitro were able to effectively inhibit the gene expression of both aurora kinases and induce apoptosis in K-562 cells, however our data clearly shown that *AURKB* proves to be a singular finding and potential prognostic biomarker that may be used as a promising therapeutic target to those patients.

## Introduction

Acute Lymphoblastic Leukaemia (ALL) is defined as a clonal expansion of an abnormal lymphocyte precursor cell, being the most common subtype of acute childhood leukemias, accounting for 75–80% of all cases^1^. Gene fusions appear as one of the most frequent alterations in and identification of ALL patients, helping to provide important prognostic information for patients, and many of these changes are associated with a high risk of relapse^2^.Therefore, chromosome translocations are a major marker of genomic instability in the pathogenesis of ALL, especially those related to the failure of chromosomal segregation or DNA repair during the cell cycle, being of fundamental importance its identification for therapeutical directions^3,4^.

The aurora kinase family are constituted of three serine/threonine kinases (AURKA, AURKB and AURKC) that acts like mitotic kinases with an important role in the regulation of the G2 / M phase of the cell cycle and several mitotic events, including centrosome duplication, mitotic spindle formation, chromosomal segregation, and cytokinesis occurring at the end of telophase, events that are essential for cell division, pointing out the importance of studying the role of these genes and their involvement in the maintenance mechanism of cell cycle stability^5,6^.

Overexpression of *AURKA* and *AURKB* genes can disrupt the normal development of cell division, increasing genetic instability, triggering the development of tumors. This abnormal expression have been well characterized in several types of aggressive cancers^7–10^.

The aim of this study was evaluate the *AURKA* and *AURKB* mRNA expression profile of pediatric ALL patients, to construct a protein–protein interaction network to evaluate the possible role of those targets in leukemogenesis pathway and the efficacy of two designed aurora kinase inhibitors in a leukemia cell line as a potential novel treatment to ALL pediatric patients.

## Results

### Non-selective Aurora Kinase inhibitors decreased cell proliferation provoking cell death and cell cycle progression modulating *AURKA*, *AURKB* and *BCR-ABL1* gene expression

Firstly, it was proved that non-selective *AURKA* and *AURKB* inhibitors GW809897X and GW806742X treatment reduced cell proliferation of leukemia cell line (K-562) with a potency constant IC50 of > 5 µM and 1.47 µM, respectively. In order to determine whether cell proliferation reduction was due to cell death process, we performed caspase 3 and 7 activity and morphological changes analysis by flow cytometry.

**Figure 1:**
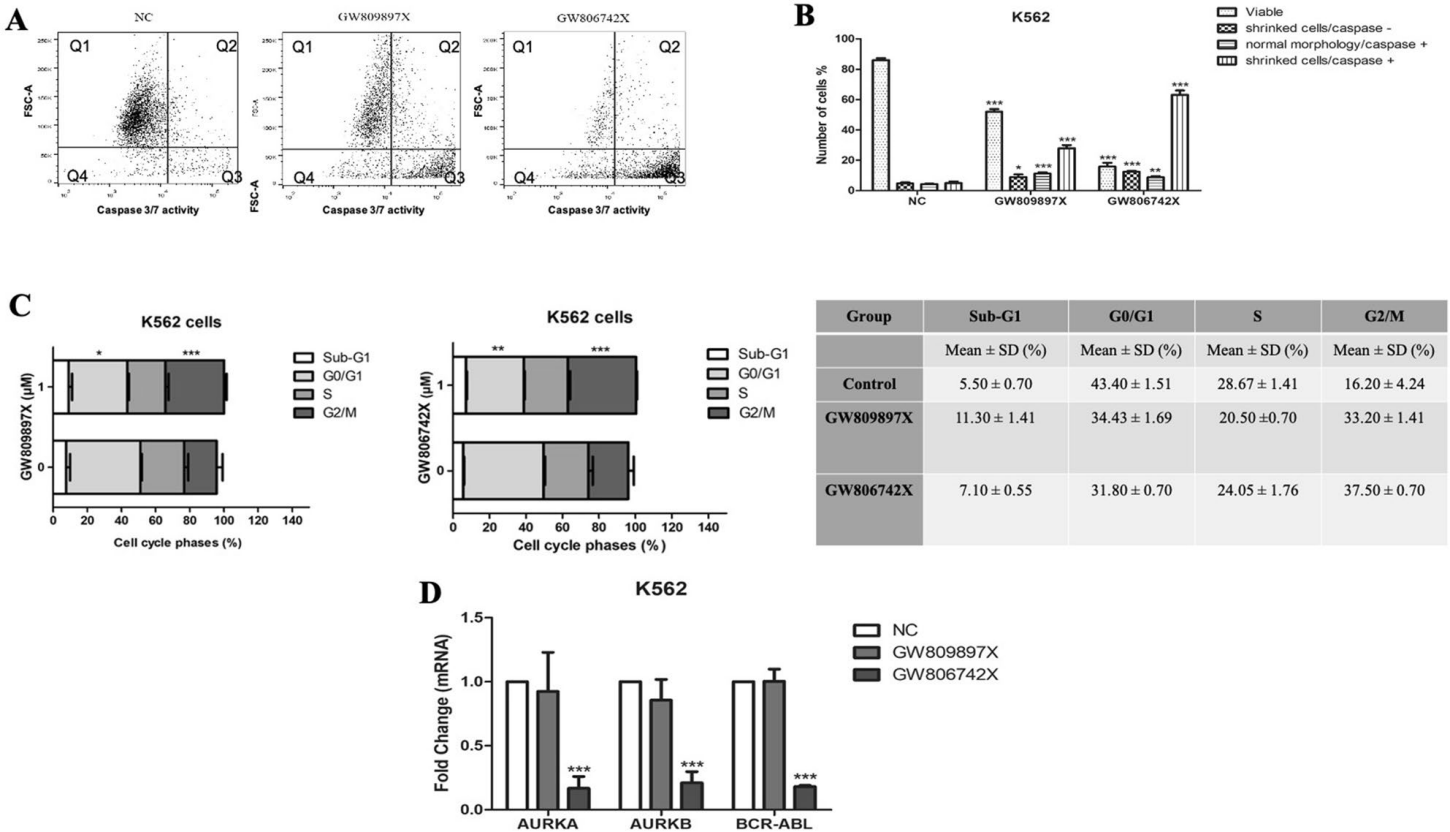
Cellular effects of AURKA and AURKB inhibitors against leukemia cell line K-562. **(A)** Dot plots are presenting the morphology analysis and caspase 3/7 activity. Q1: normal morphology/caspase negative; Q2: normal morphology/caspase positive; Q3: shrunk cells/caspase positive; Q4: shrunk cells/caspase negative. **(B)** Data of morphology and caspases represented as the mean ± standard deviation of three independent experiments. **(C)** Bar Graphs and table represents the mean ± SD of the percent of cells in Sub-G1, G_0_/G_1_, S and G_2_/M phase upon exposure to GW809897X and GW806742X. **(D)** Gene expression of *AURKA*, *AURKB,* and *BCR-ABL* after AURKA and AURKB inhibitor treatment (1 µM). significant differences: *p < 0.05; **p < 0.01; ***p < 0.001; ANOVA test and Bonferroni posttests.

Results shown in Fig. 1 demonstrated that 1 µM GW809897X and GW806742X inhibitors significantly increased the number of shrunk cells (*P* < 0.05) which indicate a possible early stage of cell death. Caspase 3 and 7 activation was also observed after the treatment (*P* < 0.0001), which confirm the apoptosis as a cell death pathway (Fig. 1A,B).

Cell cycle distribution also showed that 1 µM of both aurora kinase inhibitors induced significant G2/M phase arrest (*P* < 0.001) in k562 cell line (Fig. 1C). Compared with control group, the K-562 exposure to GW809897X and GW806742X lead to the accumulation of cells in the G2/M phase from 16.20 ± 4.24 to 33.20 ± 1.41 and 37.50 ± 0.70, respectively (Fig. 1C). Additionally, GW809897X and GW806742X also significant induced a decreased of cells in G0/G1 (*P* < 0.05 and *P* < 0.01). These data proves that GW809897X and GW806742X inhibits the cellular proliferation through G2/M cell cycle arrest.

In addition, as shown in Fig. 1D, *AURKA*, *AURKB,* and *BCR-ABL* gene expression were only affected by GW806742X (1 µM) treatment (*P* < 0.001).

### Acute lymphoblastic leukemia patients clinical features

As shown in Table 1, the 104 ALL patients included in this study were sorted into different groups by age (< 1, 1–9 and ≥ 10), white blood cells count (< 50 × 10^3^/mm^3^, 50–100 × 10^3^/mm^3^, ≥ 100 × 10^3^/mm^3^), and gender (male and female), according to their immunophenotype and chromosomal translocation. The chromosomal translocations identified in these patients were*, BCR-ABL* (15)*,E2A-PBX1* (21)*,TEL-AML1* (9)*, SIL-TAL* (4)*, MLL-AF4* (6). Chromosomal translocations could not be identified in 49 patients by RT-PCR technique performed for diagnosis.

**Table 1:** Clinical features analysis of pediatric acute limphoblastic leukemia patients.

	N (%)												
	Age				WBC count (× 10^3^)/mm^3^				Gender				
	< 1	1–9	≥ 10	^a^* p* value	< 50	50–100	> 100	*p* value	M		F		*p* value
**Immunophenotype**				0.132				0.183					0.136
Biphenotypic	0 (0)	9 (12.5)	1 (3.6)		8 (12.3)	1 (11.1)	1 (4.8)		6 (9.4)		4 (10)		
T-cell ALL	0 (0)	2 (2.8)	4 (14.3)		3 (4.6)	0 (0)	4 (19)		6 (9.4)		0 (0)		
B-cel ALL	4 (100)	61 (84.7)	23 (82.1)		54 (83.1)	8 (88.9)	16 (76.2)		52 (81.3)		36 (90)		
**Translocation**				**0.0004**				**0.013**					0.515
*BCR-ABL*	1 (25)	8 (11.1)	6 (21.4)		8 (12.3)	1 (11.1)	6 (23.8)		9 (14.1)		6 (15)		
*E2A-PBX1*	0 (0)	20 (27.8)	1 (3.6)		12 (18.5)	5 (55.6)	3 (14.3)		15 (23.4)		6 (15)		
*MLL-AF4*	3 (75)	3 (4.2)	0 (0)		3 (4.6)	0 (0)	1 (4.8)		2 (3.1)		4 (10)		
*SIL-TAL*	0 (0)	1 (1.4)	3 (10.7)		0 (0)	0 (0)	3 (14.3)		2 (3.1)		2 (5)		
*TEL-AML1*	0 (0)	8 (11.1)	1 (3.6)		8 (12.3)	1 (11.1)	0 (0)		7 (10.9)		2 (5)		
Others	0 (0)	32 (44.4)	17 (60.7)		34 (52.3)	2 (22.2)	9 (42.9)		29 (45.3)		20 (50)		

*F* Female, *M* male, *WBC count* White blood cell count.

^a^*p* value by Chi-square test; α = 0.05.

Results demonstrated significant differences between age groups considering the type of translocation (*P* < 0.0001). In these, 25% of patients with less than 1-year-old were positive for *BCR-ABL*, 29.2% patients with 1–9 years-old were positive for *E2A-PBX1* and 60.7% of patients with ≥ 10 years-old were positive for others types of chromosomal translocations.

Chromosomal translocations types frequency were also different between white blood cells count (WBC count) groups (*P* = 0.013). In the patient's group with WBC count < 50 × 10^3^/mm^3^, 18.5% were positive for *E2A-PBX1*, 12,3% for *BCR-ABL*, 12,3% for *TEL-AML1* and 52.3% had others types of chromosomal translocations that was not identified.

In the patient's group with 50 to 100 × 10^3/^mm^3^, more than half were positive for *E2A-PBX1* translocation (55.5%). Moreover, 23.8% of patients with ≥ 100 × 10^3^/mm^3^ were positive for *BCR-ABL1*, 14.3% were positive for *E2A-PBX1*, 14.3% for *SIL-TAL* and 42.8% for others translocations. Otherwise, gender was significantly different between immunophenotype groups (*P* < 0.0001). Both, male and female patients, were highly positive for B-cell ALL (81,3%) and (90%), respectively, and only male patients were positive for T-cell ALL (9,4%).

### AURKA and AURKB mRNA expression in ALL patients

**Figure 2:**
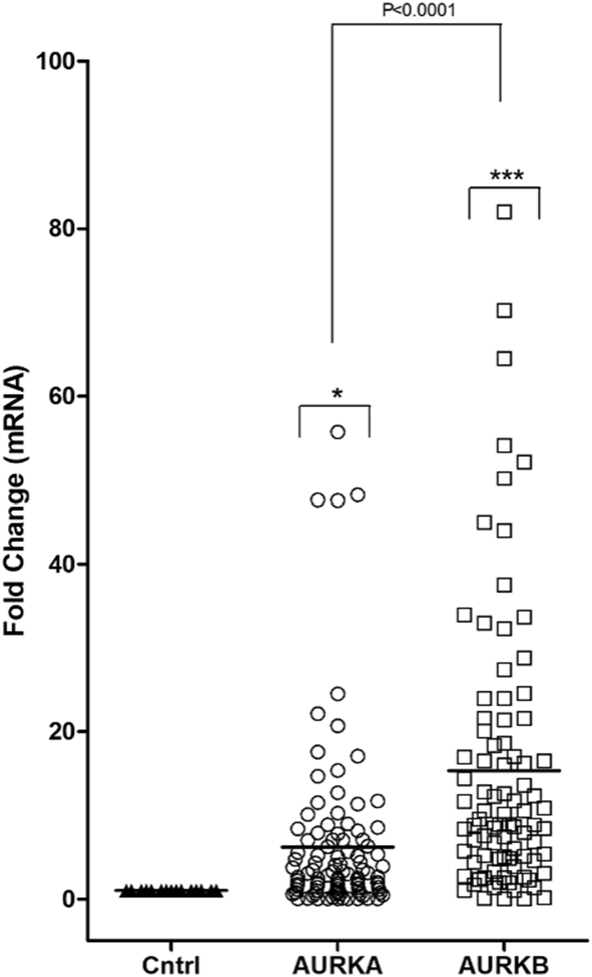
Global *AURKA* and *AURKB* expression in ALL blood samples. Fold change expression was calculated using control (Cntrl) patients as a calibrator for relative expression analysis. Data are presented as the median and each dot plot is a single patient. Kruskal–Wallis test followed by Dunn’s multiple posttests was performed. Comparison against control samples: *p < 0.05; ***p < 0.0001. *AURKB* expression is higher than *AURKB* in patients as shown in the figure (*p* < 0.001).

Since the leukemia cell line K-562 was chemosensitive for both aurora kinase inhibitors (GW809897X and GW806742X), we decided to measure the *AURKA* and *AURKB* mRNA expression in the ALL patient’s blood cells. *AURKA* was 6.20 fold change overexpressed in ALL samples compared to control samples (*P* < 0.05), while *AURKB* was 15.32 fold change overexpressed (*P* < 0.0001). Furthermore, the results demonstrated that the *AURKB* expression level was significantly higher than *AURKA* in ALL patients (*P* < 0.0001), Fig. 2.

**Figure 3:**
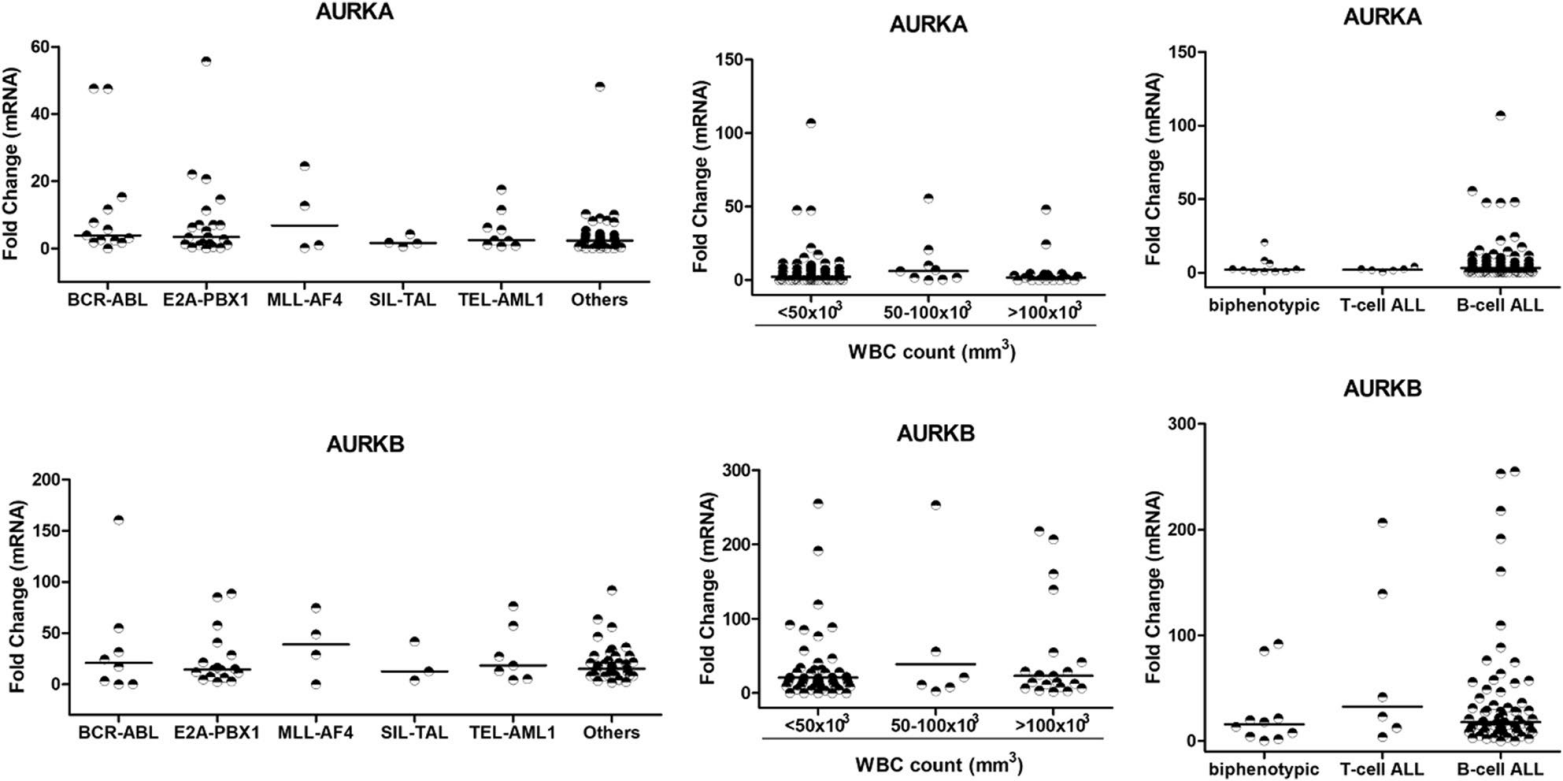
*AURKA* and *AURKB* are equally expressed among ALL patients with different translocations, white blood count or immunophenotype. Fold change expression was calculated using control (Cntrl) patients as a calibrator for relative expression analysis. Data are presented as the median and each dot plot is a single patient. Kruskal–Wallis test followed by Dunn’s multiple posttests was performed.

The next question was whether the *AURKA* and *AURKB* gene expression levels differ among the clinical features of ALL patients. As shown in Fig. 3, chromosomal translocation groups (*BCR-ABL1, E2A-PBX1, MLL-AF4, SIL-TAL, TEL-AML1*, and non-identified chromosomal translocation) were not different in terms of *AURKA* (*P* = 0.416) and *AURKB* expression (*P* = 0.948), as well as the immunophenotypic groups (biphenotypic, T-cell ALL, B-cell ALL), with no significant differences observed for *AURKA* (*P* = 0.656) and *AURKB* (*P* = 0.404). White Blood Cells (WBC) count groups were also analyzed and no significant differences were found for *AURKA* (*P* = 0.390) and *AURKB* (*P* = 0.687) expression. Moreover, no correlation was observed among WBC count and *AURKA* (Spearman r = −0.097) and *AURKB* (Spearman r = −0.062).

**Figure 4:**
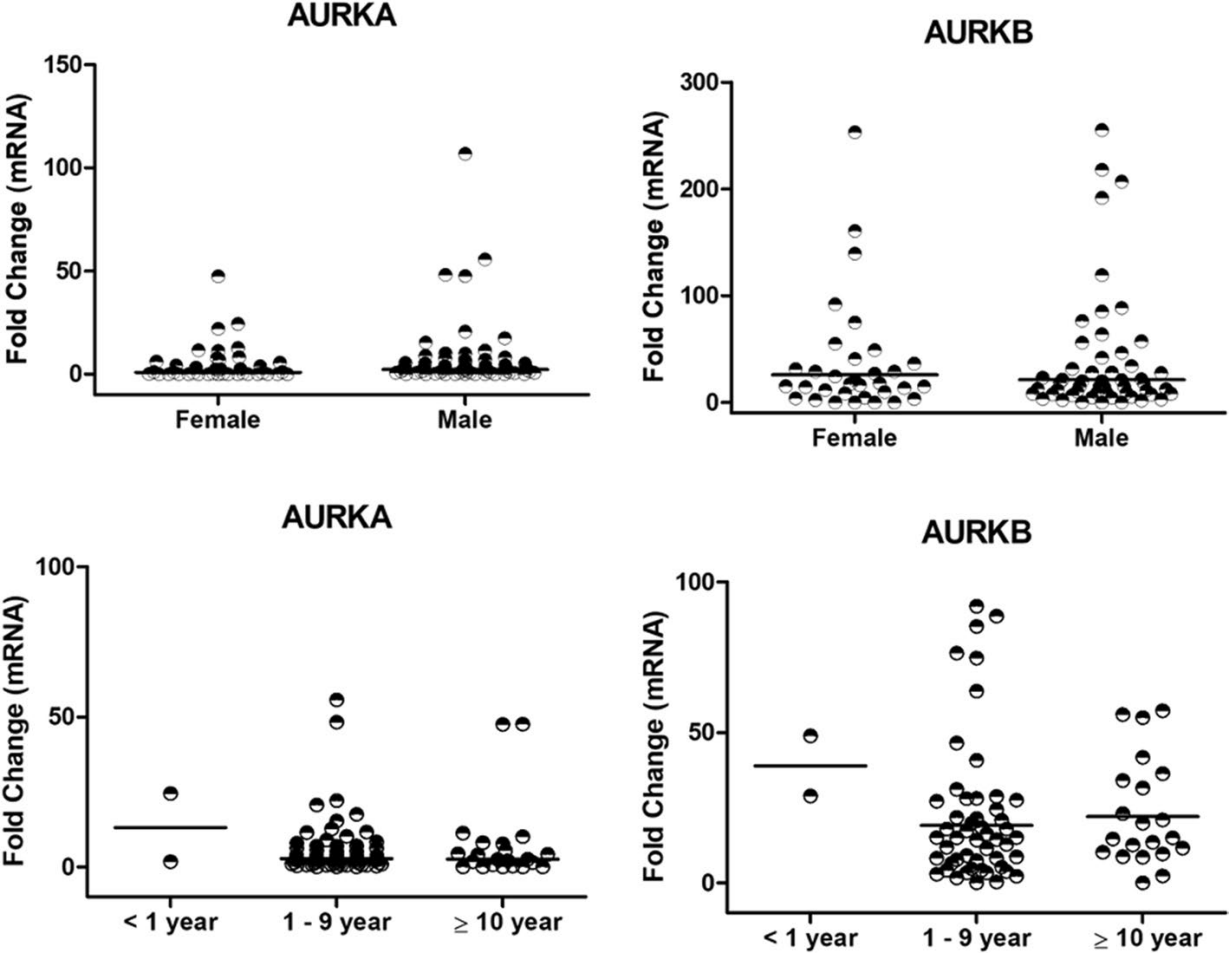
*AURKA* and *AURKB* are equally expressed considering gender and age of ALL patients. Fold change expression was calculated using control (Cntrl) patients as a calibrator for relative expression analysis. Data are presented as the median and each dot plot is a single patient. Kruskal–Wallis test followed by Dunn’s multiple posttests was performed for age and Mann–Whitney test for gender.

In Fig. 4, results showed that *AURKA* (*P* = 0.078) and *AURKB* (*P* = 0.880) expression were not different between male and female patients, as well as in the age groups which *AURKA* (*P* = 0.705) and *AURKB* (*P* = 0.635) expression levels were equally distributed.

### Patients survival rate

**Figure 5:**
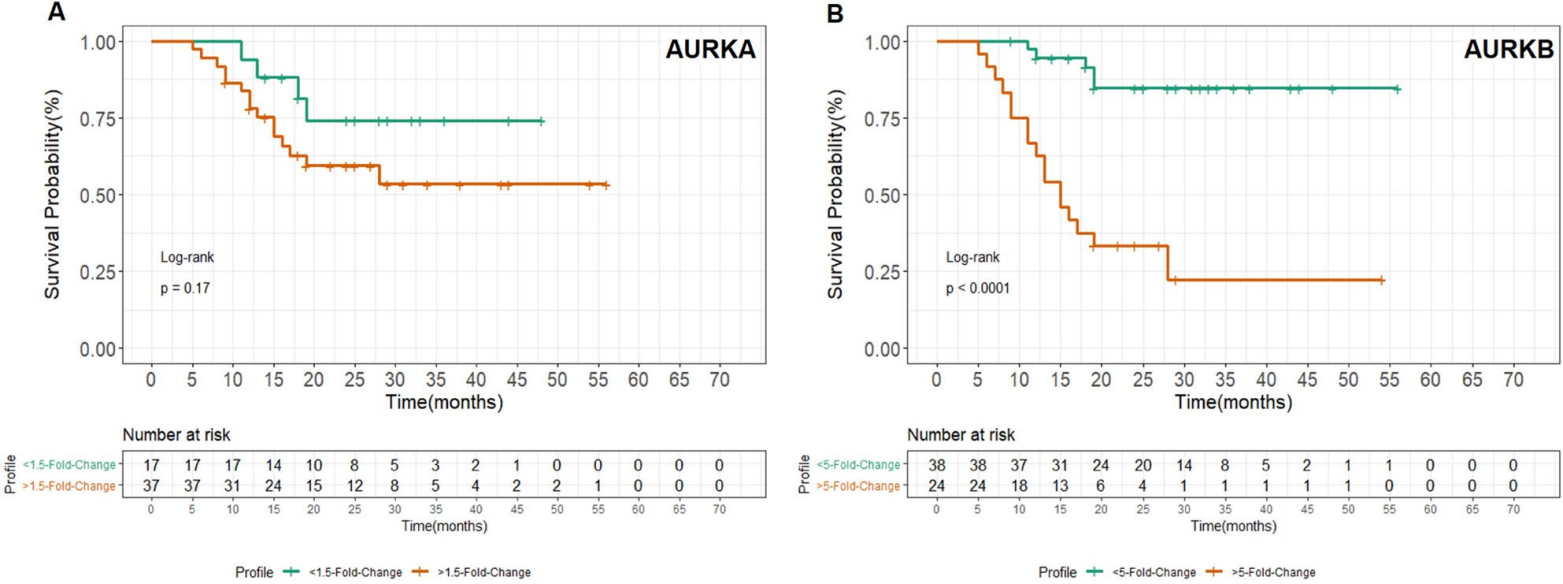
Comparison of survival time for patients with different expression levels in **(A)** *AURKA* and **(B)** *AURKB* genes with long-rank test. The survival time was statistically different between patients: *AURKA* expression level was not significant at the overall survival rates (*p* = 0.17), however *AURKB* showed a significantly lower rate survival in patients (*P* < 0.0001) that presented higher expression levels (≥ fold 5).

We also performed a patients survival overall rate to evaluate the role of *AURKA* and *AURKB* gene expression levels and its potential association with prognosis. A total of 64 patients were included, and the median time of follow-up was 22.9 months (range 5–56 months).The data was normalized and categorized assuming Log2 expression levels, for *AURKA* the expression levels was 1,fivefold and for *AURKB* fivefold. In comparison between both genes, *AURKA* did not had any impact at the patients overall survival (*P* = 0.17). However, *AURKB* expression levels predicted a poorer prognosis with significantly lower survival rates (*P* < 0.0001), as shown in Fig. 5.

### Protein–protein interaction network analysis and co-expression

**Figure 6:**
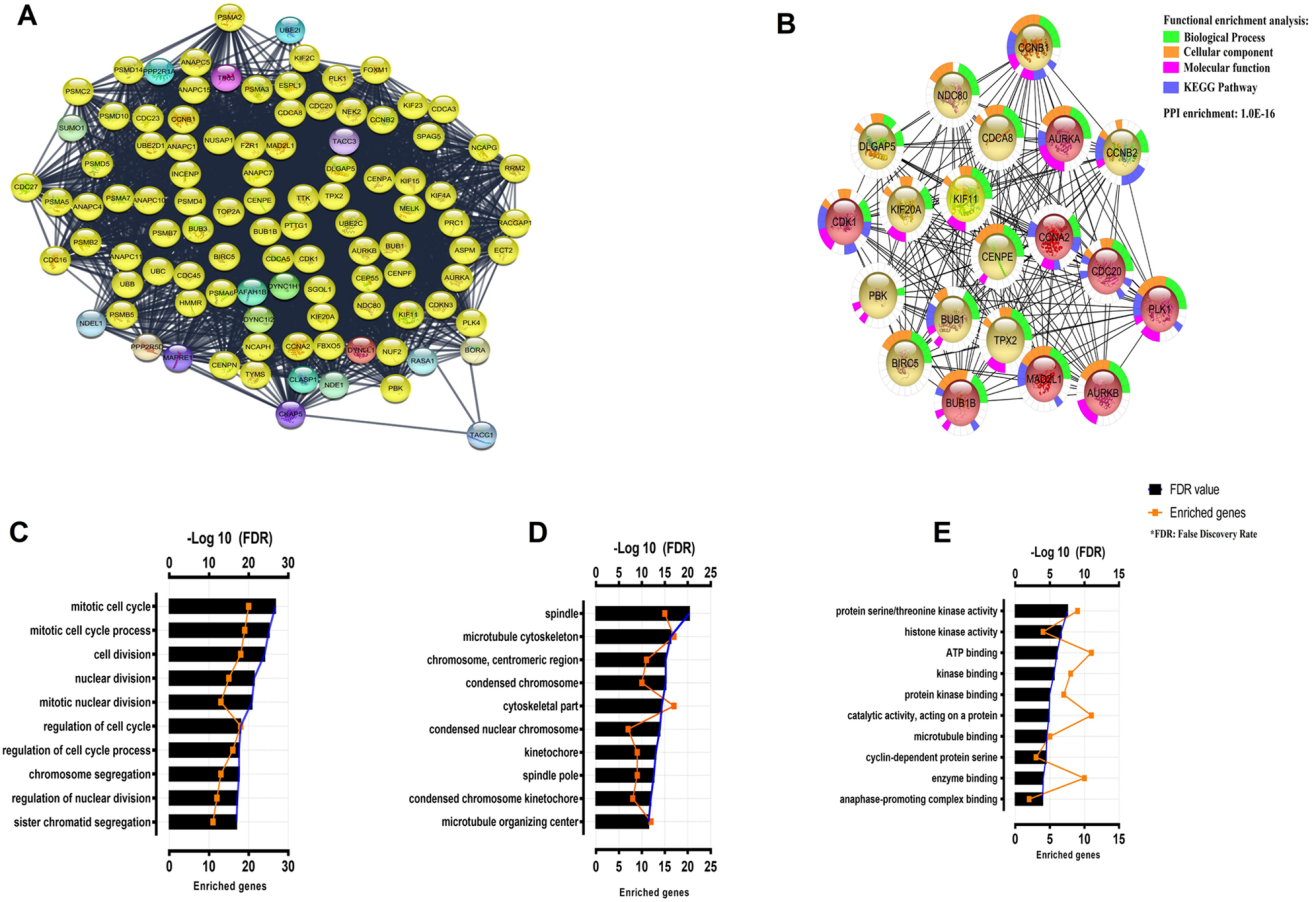
PPI network construction and module analysis. (**A**) PPI network built to provide the interaction of proteins with AURKA and AURKB using STRING, from which it was obtained with a confidence score of 0.70 consists of 102 nodes and 2,751 edges. **(B)** MCODE was used to discover closely related regions in PPI network and obtain the protein composition as shown in Fig. The module shows nodes with the closely related red color gradient and nodes with the strictly related yellow color gradient. To better predict protein function associations, network nodes were enriched with CytoHubba to produce a functional enrichment map that was converted to the Log10 scale (FDR) into the biological process (**C**), cellular component (**D**), molecular function (**E**) and KEGG pathways. *PPI* Protein–protein interaction, *FDR* false discovery rate.

To identify the high-trust hub genes, we inserted aurora kinases AURKA, AURKB into the STRING database. A Protein–Protein Interaction Network (PPI) was generated with a confidence score of 0.70 composed of 102 nodes and 2751 edges. All PPI network was analyzed using MCODE. Only the best module was chosen with a score of 53.47 (84 knots and 2219 edges). Hub genes were identified from the results of combined analysis between MCODE and CytoHubba. The PPI network had an enrichment of 1.0E−16, indicating that the proteins of these genes are biologically linked as a group. The first 20 genes identified with the MCC method were chosen by CytoHubba: *AURKA, DLGAP5, CCNB1, KIF11, NDC80, CENPE, CCNA2, BUB1B, CCNB2, MAD2L1, PLK1, TPX2, BIRC5, BUB1, AURKB, CDC20, CDK8 , PBK*. Furthermore, GO enrichment analysis showed these genes strongly associated in biological process, cellular component and molecular function, respectively for mitotic cell cycle (GO: 0000278), spindle (GO: 0005819), protein serine / threonine kinase activity (GO: 0004674), as shown in Fig. 6. The most enriched KEGG pathway was identified for cell cycle (hsa04110).

## Discussion

The advances achieved in the treatment of ALL during the last decades are a successful model in the practice of modern medicine based on translational research and clinical trials^1^. Despite the increase in cure rates, exceeding 80%, up to a quarter of the patients still present a relapse, which leads to a poor prognosis, leading to death, where there is still a high frequency rate^11^. These events are especially observed in patients who present genomic alterations that compromise the effective treatment of available chemotherapy protocols^12,13^. In this study, expression of *AURKA* and *AURKB* in ALL pediatric patients was evaluated, as well as, the efficacy of two new potential aurora kinase inhibitors as therapeutic options.

The chromosomal translocations identified in the 104 patients analyzed were *E2A-PBX1* (20.19%), *BCR-ABL1* (14.42%), *TEL-AML1* (8.65%), *SIL-TAL* (3.8%), *MLL-AF4* (5.8%), these gene alterations were previously described in other studies in Brazilian ALL pediatric population^14–17^. However, in our study it was noted a higher prevalence, among the identified gene fusions, the types *E2A-PBX1* and *BCR-ABL*. Is widely known in the literature that gene fusions as *E2A-PBX1*, *BCR-ABL1* appears to have the worst outcome and prognosis compared to the others types, pointing an emerging need to new therapeutic approaches and targets specially to these patients^18–23^.

The ALL pediatric patients analyzed in this study showed overexpression of both, *AURKA* and *AURKB* genes, showing that these genes seems to have a prominent importance in this disease model, regardless of clinical findings and variables of risk management. However, the *AURKB* stands out as significantly relevant when compared to *AURKA*. This is the first study in Brazil to show *AURKA* and *AURKB* expression data in pediatric patients with acute lymphoid leukemia, as well as to present them in comparison to clinical parameters. It was important to notice that no clinical features, presented by patients, was able to differentially influence analysis of *AURKA* and *AURKB* gene expression, showing the importance of aurora kinases as biomarkers in pediatric ALL pathogenesis.

The overall survival rate demonstrated that *AURKA* did not show significant results at the patient’s overall survival (*P* = 0.17). Nevertheless, *AURKB* expression levels predicted a poorer prognosis with significantly lower survival rates (*P* < 0.0001). In hematological malignancies, *AURKA* and *AURKB* overexpression are found in patients with cytogenetic abnormalities that are unfavorable to the prognosis and which compromise patients' survival^24^. These changes have been well described in acute myeloid leukemia^25,26^, Myelodysplastic Syndrome^27–29^, Chronic Myeloid Leukemia^30^ and also in the ALL^31^.

In K-562 cells, both designed inhibitors, GW809897X and GW806742X, reduced cell proliferation, cell cycle progression and potentially induced apoptosis. However, only GW806742X inhibitor was able to strongly reduced AURKA and AURKB activity, and the molecular signature, of K-562 cells, *BCR-ABL* as well. As described in the literature, the overall rates and prognosis of ALL patients with *BCR-ABL* (Ph+) are very poor^32,33^, and aurora kinase inhibitors provided a new option for future clinical and therapeutic options for these individuals^34^. Ikezoe et al. (2007) showed that the K-562 cell line, from a diverse panel of 15 leukaemia strains, presented the highest expression of the *AURKA* and *AURKB*, proving the efficacy of using this particular experimental model to study potential inhibitors against these targets, as shown in this study^35^.

Cells with overexpression of *AURKA* overlap mitotic spindle control and enter anaphase despite abnormal spindle formation^36^. The overexpression of an inactive form of *AURKB* in cells would also compromise cell cycle control points and spindle formation because *AURKB* activity is required for recruitment of cycle check proteins^24,37^. Studies have shown that in murine models the overexpression of *AURKB* induce tetraploidy^38,39^, and cytokinesis failure in the absence of proper functioning of *AURKB* activity is a good explanation of why downregulation causes an increase in ploidy, and many studies supported the classification of *AURKB* as a cancer promoting gene^40,41^.

Cell cycle proteins have been show as potential anticancer targets^42^. To identify AURKA and AURKB interaction pathways in ALL pathogenesis, a PPI network was constructed, which could evidence the presence of both, and their participation in protein expression modulation of essential pathways for cellular organization during division, highlighting the mitotic cell cycle pathway and spindle control that appears with high correlation in this interaction pathway, demonstrating the importance of these proteins in mitotic organization. It’s important to emphasize that further studies to comprehend the role of AURKA and AURKB modulation in mitotic cell cycle pathway and spindle control might be performed for a better understanding of AURKA and AURKB in this scenario of ALL pathogenesis, which are highly related to key activities in the pathogenesis and origin of chromosomal translocations that are the molecular signature presented in this disease^43–45^.

The presence of *AURKB* overexpression in ALL patients described in this study, proves to be an important target to inhibition by specific target molecules, as GW806742X. It was shown, that AURKB inhibitors interferes with normal chromosome alignment during mitosis and induces endoreduplication, leading cells to death through catastrophic mitosis, becoming a suitable anticancer strategy^37,46,47^. In specific cases, inhibitors of disease-related aurora-kinases have been used experimentally with some success and mark a major advance in the treatment of patients with ALL^6,48^ .

The aurora kinase inhibitors tested in vitro*,* were able to effectively induce apoptosis of K562 cells, but only GW806742X was able to inhibit the gene expression of the *AURKA* and *AURKB* as well. The results indicate that the overexpression of the *AURKA* and *AURKB* genes is an important finding in childhood ALL in this study, despite the clinical features. However is clearly shown that *AURKB* proves to be a singular molecular finding in this population, strongly supported by its expression associated with poorer survival rates, pointing out that *AURKB* may be used potentially as a prognostic biomarker and therapeutic target for pediatric ALL patients.

## Material and methods

### Patients and samples

A total of 104 ALL pediatric patients were included in this study, all diagnosed at Octávio Lobo Children’s Hospital (Belém-Brazil) according to the French-American British (FAB) criteria^49^. The clinical data from patients were analyzed based on risk-stratification criteria of Berlin-Franklin-Münster (BFM)^50^. Blood samples were collected at the time of diagnosis and samples from 40 healthy volunteers were used as healthy control. The study was approved by the Ethics Committee of the Ophir Loyola Hospital (approval number: 119.649), informed written assent was obtained from the patient’s legal guardians and all methods were carried out in accordance with Helsinki guidelines and regulations.

### Cell culture

Leukemic cell line K-562 were kindly provided by Dr. Vivian Rumjanek from Federal University of Rio de Janeiro. Cells were cultured in RPMI 1640 medium supplemented with 10% fetal bovine serum (FBS) and 1% penicillin–streptomycin at 37 °C in a 5% CO_2_ air-humidified atmosphere at 37ºC and cell confluence was observed in conventional microscope.

### AURKA and AURKB drug inhibitors

Aurora Kinase (AURKA and AURKB) inhibitors GW809897X and GW806742X, belonging to the chemotype 16:2,4-diamino-pyrimidines, was obtained from a panel provided by Dr. Bill Zuercher from University of North Carolina and the Structural Genomics Consortium. Both inhibitors were well characterized as a molecular probe for targeted therapy studies by Elkins and co-workers (2016)^13^.

### Alamar blue assay

Alamar Blue assay is a classic fluorometric dye-based cytotoxicity method well established in the literature^51^. K-562 cells were seed at a concentration of 5 × 10^3^ cells per well in 96-well plate and incubated for 24 h for stabilization. Then, cells were treated with the concentration curve of inhibitors GW809897X and GW806742X in a final volume of 200 µL. Negative control was treated with drug solvent DMSO (0.01%). After 72 h of exposure, Alamar blue reagent was added and incubated for 3 h. Finally, fluorescence was measured by Beckman Coulter Microplate Reader DTX 880 (Beckman Coulter, USA) with an excitation length wave of 560 and emission length wave of 590.

### Caspase 3 and 7 activity

Cells were seed (5 × 10^3^ cells per well) in a 96-well plate and treated with 1 µM of inhibitors GW809897X and GW806742X for 72 h. After the exposure time, cells were washed with PBS and incubated with 5 μM of CellEvent Caspase-3/7 (ThermoFisher, USA) reagent detection in PSB with 5% serum for 30 min. Thereafter, caspase 3/7 activity was measured by fluorescence intensity analysis in a BD FACSVerse (Becton–Dickinson, USA) cytometer. Cells in the apoptosis process exhibit bright green fluorescence and normal/viable cells exhibit minimal fluorescence.

### Cell cycle progression

To evaluate cell cycle distribution, K-562 cells were seeded at 3 × 10^3^ cells per well in 96-well plate and treated with a 1 μM concentration of GW809897X and GW806742X for 72 h of exposure. After this, cells were fixated in 80% ethanol solution for 30 min at 4 °C and then incubated with propidium iodide (50 μg/mL) for 15–30 min. A total of 10,000 events were evaluated by flow cytometry (BD FACSVerse) and data were analyzed using FlowJoSoftware v.10.

### RNA extraction and cDNA synthesis

RNA from patients’ blood samples, healthy volunteers and from K-562 cell line were extracted using TRIzol Reagent (Life Technologies, Foster City, CA, USA) according to manufacturer’s instructions. The RNA samples were reversely transcribed to cDNA using *High Capacity cDNA Reverse Transcriptase* ((Life Technologies, Foster City, CA, USA) in a Verity PCR System thermal cycler (Applied Biosystems, USA). cDNA samples were stored at − 20 °C until the tests were performed.

### Quantitative real-time PCR (qPCR)

The genes selected for expression analysis in patients and in cell lines were *AURKA* (Hs01582072_m1) and *AURKB* (Hs00945858_g1) and *GAPDH* (Hs 02786624_g1) was used as an internal control. Such genes are commercially available as TaqMan Gene Expression Assays (Life Technologies, Foster City, CA, USA). qPCR was performed using *QuantStudio5 Real-Time PCR* system (Applied Biosystems, USA). For each sample was used concentrations as following: 3 μL of cDNA, 1 μL of each primer/probe, 12.5 μL of *TaqMan* Gene Expression Master Mix (Life Technologies, Foster City, CA, USA) and 8.5 μL of Ultra-pure H_2_O.

Each assay was performed at least three times according to *Minimum Information for Publication of Quantitative Real-Time PCR Experiments- MIQE Guidelines*^52^. The gene expression levels were based on absolute and relative analyses and calculated using the 2—^ΔΔCT^ (delta-delta threshold cycle) method, the expression level of the gene of interest is reported relative to the reference gene for each sample^53^.

### PPI network building and module analysis

*AURKA* and *AURKB* genes were submitted to the STRING Protein Data Retrieval Search Tool (v11.0) (http://string-db.org/)^54^. The confidence score was 0.70, with the other parameters being used as standard. The maximum number of 100 interactions has been tested. The Molecular Complex Detection (MCODE) application was used to analyze the PPI (Protein–Protein Interaction) network module and MCODE scores > 50 and the number of nodes > 80 were set as cutoff criteria with the default parameters (Degree cutoff ≥ 2, Node score cutoff ≥ 2, K-core ≥ 2 and Max depth = 100)^55^. Finally, the CytoHubba plugin was used to explore PPI network hub genes with the Maximal Click Centrality (MCC) metric for better PPI network performance^56^. Program STRING enrichment was used for GO (Gene Ontology) and KEGG enrichment, and Cytoscape (v3.7.1) was used to view PPI and co-expression networks^57^.

### Statistical analysis

Three independent experiments were performed in triplicate. All data were expressed as Mean or median ± dispersion measures depending on the normality of samples. For it, the Shapiro–Wilk test was applied to determine if samples followed a normal distribution. Comparison tests were performed to compare two or more different groups using Analysis of Variance (ANOVA), Bonferroni posttest and t-test or their corresponding non-parametric tests. Frequency data were analyzed by Chi-square test and correlation analysis was performed using the Spearman test. To predict the overall survival rate from patients log-rank test was calculated from the date of diagnosis to the date of mortality or last follow-up, using R package software^58^. Significant differences were determined by setting a significant level in *P* < 0.05 (confidence interval of 95%).
